# Analysis of the energy consumption of private households in Germany using multi-level cross-impact balance approach - Data

**DOI:** 10.1016/j.dib.2016.12.037

**Published:** 2016-12-21

**Authors:** Stefan Vögele, Patrick Hansen, Witold-Roger Poganietz, Sigrid Prehofer, Wolfgang Weimer-Jehle

**Affiliations:** a Forschungszentrum Jülich, Institute of Energy and Climate Research – Systems Analysis and Technology Evaluation (IEK-STE), 52425 Jülich, Germany; b Karlsruhe Institute of Technology, Institute for Technology Assessment and Systems Analysis (ITAS), 76021 Karlsruhe, Germany; c Stuttgart Research Center for Interdisciplinary Risk and Innovation Studies (ZIRIUS) University Stuttgart, 70174 Stuttgart, Germany

## Abstract

Many studies stress the needs of interdependence analysis under the special conditions of multidisciplinary systems that include social systems. This applies, in particular, to scenarios on future energy demand and supply. Using the example of the residential sector in Germany we provide information on factors and their possible outcomes taking multidisciplinary aspects into account. In addition, futures are presented reflecting consistent combinations of the outcomes of the selected factors. These futures can be used as storylines for further analyses (see (S. Vögele, P. Hansen, W-R. Poganietz, S. Prehofer, W. Weimer-Jehle) [1]).

**Specifications Table**

**Table:** 

Subject area	*Economics, Social Sciences*
More specific subject area	*Energy Economics, Energy Policy*
Type of data	*Tables, figures*
How data was acquired	*Survey*
Data format	*Raw, analyzed*
Experimental factors	*Selection of interdisciplinary group of experts with knowledge on energy policy, energy supply and demand*
Experimental features	*Expert interviews*
Data source location	*Germany*
Data accessibility	*Data is provided in*Supplementary materials*directly with this article*

**Value of the data**

• The data describes lists of factors and their links which may be directly or indirectly relevant for the future of the residential sector in Germany.
• Beside information on factors on a sector-specific level data on factors and their interactions on national and international level are provided.
• Using a Cross-Impact Balance approach consistent context scenarios can be identified.
• This data can be used to evaluate consistencies of scenarios for the residential sector in Germany.
• The data can also be used for the specification of storylines for other research fields.

## 1 Data

Factors and their possible outcomes which may be directly or indirectly relevant for the energy consumption of private households in Germany were collected by asking an interdisciplinary group of experts. In a second step the group of experts specified the interactions among the outcomes on a semi-quantitative manner by evaluating the direct influence each outcome has on another one. The factors are subdivided in sectoral, national and international factors for reducing the efforts needed for specifying the links between the factors and to enable the use of the identified network of causes and effects in a Cross-Impact Balance (CIB) framework (see e.g. [2], [3]).

Beside the lists of factors and outcomes, information on possible consistent futures is provided (see supplementary data) whereas a future is defined as consistent if for each factor the selected outcome is supported at least as strongly as any other outcome of the corresponding factor. If another outcome dominates the selected one, this will be considered to be a contradiction between the assumed set of outcomes.

## 2 Experimental design, materials and methods

The information on the factors and their links was collected by asking a group of three economists, four engineers and three experts with a background as political scientists. All experts are employed at the Institute of Energy and Climate Research at the Forschungszentrum Jülich. Focusing on the participation of researchers of this institute facilitated the implementation of several time-consuming inquiry rounds.

For each scale the experts nominated descriptors first. After reducing the lists to a manageable size, the experts judged together the strengths of impacts among possible factor specific outcomes evaluating the direct influence of each outcome on another one. Strong positive influences are indicated by using “+3”, strong negative by using “-3” and lower influences by using “+2”, “+1”, “0”, “−2” and “−1”, respectively. Using the software SzenarioWizard 4.11 [4] the resulting matrices of interactions were analyzed with respect to consistency: For each set of outcomes the balance of all incoming impacts on the outcome were added up. If each outcome of the selected set is supported at least as strong as any other outcome of the corresponding factor the selected set is defined as consistent.

Based on the selected consistent outcome sets possible future are defined which can be used as storylines for further analyses (e.g. by using bottom-up models) (see [1]).

## Footnotes

Appendix A Supplementary data associated with this article can be found in the online version at http://dx.doi.org/10.1016/j.dib.2016.12.037.
