# Supplementary material for: Analysis of the energy consumption of private households in Germany using multi-level cross-impact balance approach - Data
Source: Data Brief. 2016 Dec 21;10:515–7. doi: 10.1016/j.dib.2016.12.037 (PMC5199154; doi:10.1016/j.dib.2016.12.037)
Supplement: Supplementary file 1 — Supplementary material [file mmc1.docx]

Table 1. Influencing factors on the global level

| **Influencing factor** | | **Possible Outcomes** | |
| --- | --- | --- | --- |
| **Factor** | **Remarks** |  | **Indicative Values/Explanation** |
| Growth of GDP (global) | Average growth rate | - slightly increasing | 2010-2030: ~3%/year |
|  |  | - strongly increasing | 2010-2030: ~3.5%/year |
| Oil price | Price for one barrel of brent oil | - low | 2030: ~100$/bbl |
|  |  | - moderate | 2030: ~125$/bbl |
|  |  | - high | 2030: ~175$/bbl |
| Population (EU) | Annual growth rate for population in EU 28 | - increasing | ~ 0.1%/year |
|  |  | - decreasing | ~ -0.4%/year |
| International climate change policy |  | - coordinated and ambitious, EU forerunner |  |
|  |  | - not coordinated, less ambitious |  |
| CO_2_-reduction EU | Reduction in Greenhouse gases measured as CO_2_ (EU 28) | - CO_2_ reduction targets are missed, no new targets | reduction of not more than 20% below 1990 levels by 2030 |
|  |  | - moderate CO_2_ red. targets | reduction of 30% CO_2_  below 1990 levels by 2030 |
|  |  | - ambitious CO_2_ reduction targets | reduction of 40% CO_2_below 1990 levels by 2030 and 80% by 2050 |
| Price for CO_2_-allowances |  | - low | <30 Euro/ton CO_2_ |
|  |  | - moderate | 30-50 Euro/ton CO_2_ |
|  |  | - high | 50-75Euro/ton CO_2_ |
|  |  | - very high | 75-100Euro/ton CO_2_ |
| Environmental protection | Attitude to measures for environmental protection | - low rate | nearly the same level as today |
|  |  | - high rate |  |
| Willingness to invest |  | - low | nearly the same level as today |
|  |  | - high |  |
| Energy res.: scarcity | Scarcity of oil and gas | - new deposits |  |
|  |  | - increasing scarcity |  |
| Energy res. Trade | Limitations on the trade with oil and gas | - free | nearly the same level as today |
|  |  | - restrained | new trade barriers |
| Other res.: scarcity | Scarcity of resources like | - new deposits |  |
|  |  | - increasing scarcity |  |
| Other res.: trade |  | - free | nearly the same level as today |
|  |  | - restrained | new trade barriers |
| Desertec and similar supranational energy projects | Supranational energy projects which are aimed for increasing energy trade within Europe and between Europe and other regions | - not put into action |  |
|  |  | - put into action with delay |  |
|  |  | - put into action as planned |  |
| Electricity demand EU | Demand for electricity in EU-28 | - decreasing | ~ -0.2 %/year |
|  |  | - constant | ~ 0.2 %/year |
|  |  | - increasing | ~ 0.6 %/year |
| Energy cons. (World) |  | - slight increase | ~1.0%/year |
|  |  | - moderate increase | ~1.2%/year |
|  |  | - strong increase | ~1.5%/year |

Table 2. Influencing factors on the national level

| **Influencing factor** | | **Possible outcomes** | |
| --- | --- | --- | --- |
| **Factor** | **Remarks** | **Outcome** | **Indicative Values/Explanation** |
| Growth of GDP (Germany) | annual change in gross domestic product | - minor increase | approx. 0.5% per year |
|  |  | - strong increase | approx. 1% per year |
| Oil price | Price for oil on the international market | - moderate | 2030:~125$/bbl |
|  |  | - high | 2030: ~175$/bbl |
| Population | number of persons in Germany | - slight decrease | 2030: 79 million |
|  |  | - strong decrease | 2030: 77 million |
| Climate and energy policy (national) | Focus of climate and energy policy in Germany | - focus on energy security | reduction of energy demand and diversification of energy sources |
|  |  | - focus moderate greenhouse gas red. | CO_2_ reduction target: -40 % (2030) |
|  |  | - focus ambit. greenhouse gas red. | CO_2_ reduction target: -60 % (2030) |
| Environmental awareness |  | - unchanged | situation as it is today |
|  |  | - increasing | more interest in “green” lifestyle and “green” economy |
| Knowledge generation |  | - slightly increasing | continuation of current trends |
|  |  | - strongly increasing | acceleration of the current development |
| Innovation dynamics | development and deployment of new techniques | - constant | continuation of current trends |
|  |  | - increasing | acceleration of the current development |
| Willingness to invest |  | - low | continuation of current trends |
|  |  | - high | acceleration of the current development |
| Replacement of assets |  | - slightly accelerated | continuation of current trends |
|  |  | - accelerated significantly | acceleration of the current development |
| Climate change | Visibility of impacts of climate change in Germany | - slightly visible | only small changes in temperature |
|  |  | - clearly visible | higher temp. in winter, heat waves in summer |
| Resource scarcity | Scarcity of energy resources | - increasing | continuation of current trends |
|  |  | - strongly increasing | higher increase in energy demand than tapping new deposits |
| Space requirements | average space requirement person (private households) | - slight increase | ~ 46 m^2^ per person |
|  |  | - strong increase | ~ 50 m^2^ per person |
| Equipment with electric devices (priv. househ.) | Number of electric application in the sector private households | - increasing | continuation of current trends |
|  |  | - strong increase | acceleration of the current development |
| Energy demand: Private households | Demand of the private households for electricity and heat | - gradual decline | ~ -1% per year |
|  |  | - strong decline | ~ -1.5% per year |
| Energy demand: Industry | Demand of the industry for fuels | - slight increase | ~ 0.5% per year |
|  |  | - gradual decline | ~ -0.5% per year |
|  |  | - strong decline | ~ -1% per year |
| Energy demand: Transport | Demand of fuels for transportation purposes | - gradual decline | ~ -0.5% per year |
|  |  | - strong decline | ~ -1% per year |
| Energy demand: Others (Business, trade, …) |  | - gradual decline | ~ -0.5% per year |
|  |  | - strong decline | ~ -1% per year |
| Expansion of electricity grid | Expansion of the high-voltage grid | - restrained | lower the proposed by [[1](#_ENREF_1)] |
|  |  | - unrestrained | as proposed by [[1](#_ENREF_1)] |
| Degree of centralization | Concentration of electricity production on selected sites | - low | less energy supply hotspots than today |
|  |  | - high | energy supply hotspots as today |
| Size of energy sup. Units | Average size of units producing electricity | - increased application of small units | significant more energy supply units with less than 1 MW |
|  |  | - dominant application of large units |  |
| Regional level of diversification | Regional distribution of population | - constant | situation as it is today |
|  |  | - increased | increase in the differences between regions |

References:

[1] dena. dena Grid Study II (dena-Netzstudie II). Berlin: Deutsche Energie-Agentur GmbH (dena), 2010.

Table 3. Influencing factors on the sectoral level

| **Influencing factor** | | **Possible outcomes** | |
| --- | --- | --- | --- |
| **Factor** | **Remark** | **Outcome** | **Indicative Values/Explanation** |
| Growth of GDP (Germany) | annual change in gross domestic product | - minor increase | approx. 0.5%/ year |
|  |  | - strong increase | approx. 1%/ year |
| Fuel prices | Prices for fuels the households have to pay | - strong increase | 3,0 %/year |
|  |  | - moderate increase | 1.5 %/year |
|  |  | - minor increase | 1.0 %/year |
| Disposal income | income without expenditure for energy | - slight decrease | - 0.3 %/year |
|  |  | - strong decrease | - 0.7 %/year |
| Population | number of persons living in Germany | - slight decrease | 2030: 79 million |
|  |  | - strong decrease | 2030: 77 million |
| Use of decentr. energy supply options | renewable energies for space heating and hot water (growth rate) | - strong increase | 12.5 %/year |
|  |  | - moderate increase | 10.0 %/year |
|  |  | - slight increase | 7.5 %/year |
| Use of renewables | renewable energies for space heating and hot water (relative share) | - strong increase | 2030: 50 % |
|  |  | - moderate increase | 2030: 30 % |
|  |  | - slight increase | 2030: < 30 % |
| Working population | number of the national working population | - increasing | 2030: > 43 million |
|  |  | - constant | 2030: 43 million |
|  |  | - decreasing | 2030: 42 million |
| Relevance of households with elderly persons | change of number of households with elderly persons compared to today | - increasing | 2030 + 20 % |
|  |  | - constant | 2030: constant (compared to today) |
|  |  | - decreasing | 2030: - 20 % |
| Expansion of electricity grid | expansion of the high-voltage grid | - restrained | less than half of DENA II study |
|  |  | - unrestrained | as proposed by DENA II study |
| Regional level of diversification | regional distribution of population | - increasing | increase in the differences between regions |
|  |  | - constant | situation as it is today |
| Climate change/energy policy | focus of climate and energy policy in Germany | - focus ambit. greenhouse gas red. | CO_2_ reduction target: -60 % (2030) |
|  |  | - focus moderate greenhouse gas red. | CO_2_ reduction target: -40 % (2030) |
|  |  | - focus on economics | moderate reduction of energy demand |
| Innovation dynamics | development and deployment of new techniques | - strong increase | 2.0 %/year |
|  |  | - slight increase | 1.0 %/year |
|  |  | - constant | <1.0 %/year |
| Energy performance of buildings | high means low energy demand and low corresponds to a high energy demand. | - High | <100 kWh/(m^2^a) |
|  |  | - Medium | 100-140 kWh/(m^2^a) |
|  |  | - Low | >140 kWh/(m^2^a) |
| Rental charge/price of buildings and flats |  | - strong increase | 2.5 %/year |
|  |  | - slight increase | 1.5 %/year |
| Oil price | Price for oil on the international market | - high | 2030: ~175$/bbl |
|  |  | - moderate | 2030: ~125$/bbl |
|  |  | - low | 2030: ~100$/bbl |
| Final energy demand | demand for energy of the end-users | - gradual decline | ~ -1% per year |
|  |  | - strong decline | ~ -1.5% per year |

Abbreviation used str.: strong, incr.: increasing, decr.: decreasing, act.: action, dep.: deposits, scar.: scarcity, mod.: moderate, coord.: coordinated

Remark: * outcome represented most frequently in the corresponding group of scenarios

Fig. A-1. Consistent Scenarios on Global Level and identified “Global Futures”

Fig. A-2. Consistent Scenarios on National Level and identified “National Futures”

Fig. A-3. Consistent Scenarios on Sectoral Level and identified “Sectoral Futures
